# Coarse Alignment of Marine Strapdown INS Based on the Trajectory Fitting of Gravity Movement in the Inertial Space

**DOI:** 10.3390/s16101714

**Published:** 2016-10-15

**Authors:** Lin Zhao, Dongxue Guan, Jianhua Cheng, Xiaomin Xu, Zaihui Fei

**Affiliations:** Marine Navigation Research Institute, College of Automation, Harbin Engineering University, Harbin 150001, China; zhaolin@hrbeu.edu.cn (L.Z.); xuxiaomin@hrbeu.edu.cn (X.X.); feizaihui@hrbeu.edu.cn (Z.F.)

**Keywords:** coarse alignment, Inertial Navigation System (INS), gravity vector, inertial frame, marine applications, least-squares fitting of spatial circle

## Abstract

A ship experiences the random motion of sea waves during its travels. Hence, the coarse alignment of the marine strapdown Inertial Navigation System (INS) suffers from rocking disturbances such as pitch and roll. In this paper, a novel approach of marine coarse alignment was proposed for avoiding the resulting loss of accuracy from rocking disturbances. Unlike several current techniques, our alignment scheme is intuitional and concise. Moreover, the coarse alignment can be implemented without any external information. The gravity vector and its derivative expressed within the inertial frame can describe the attitude matrix between an inertial frame and the local geographic frame. We address the challenge of calculating the gravity derivative by the least-squares fitting of the trajectory of the gravity movement in the inertial frame. Meanwhile, the integration of angular rates measured by gyroscopes allows one to compute the attitude matrix between the inertial frame and the body frame. The coarse alignment can be thus accomplished by the combination of the above two attitude matrices. The experimental results show that the coarse alignment is effective with high accuracy and stability for demanding marine applications.

## 1. Introduction

The initial alignment of a strapdown Inertial Navigation System (INS) is to obtain initial attitude information referring to pitch, roll, and heading angles [1]. Generally, a coarse alignment and a subsequent fine alignment complete the initial alignment. The purpose of coarse alignment is to estimate the coarse attitude in a few minutes, which initialize the fine alignment. The error of coarse attitude is required to be limited to about one degree so that the final alignment time and accuracy can be satisfactory.

The typical initial alignment is realized by using two pairs of non-collinear vectors [2,3,4], which are suitable for stationary strapdown INS. In strapdown INS, the Inertial Measurement Unit (IMU) consists of three accelerometers and three gyroscopes. When the INS is stationary, the accelerometers and gyroscopes are used to measure the gravity and earth rotation velocity in the body frame, respectively. The initial attitude matrix can then be determined by using two pairs of non-collinear vectors; essentially, it is a Wahba’s problem [5,6]. One pair of the non-collinear vectors is the gravity and earth rotation velocity in the body frame (see all frame definitions in Section 2), namely the measurements from IMU. Another pair of the non-collinear vectors is the gravity and earth rotation velocity in the navigation frame, which can be calculated by using local latitude information. However, for marine strapdown INS, the ship continuously suffers from pitch and roll motions caused by sea waves and wind. The ship’s angular velocity is consequently high, several orders of magnitude greater than the Earth rotation velocity. That is to say, we cannot obtain the earth rotation velocity from the gyroscopes measurements. Therefore, this typical coarse alignment approach is not applicable for marine strapdown INS in this case.

To address the above problem, a novel alignment approach [7] abandoning the direct use of the earth rotation velocity was proposed in the year 2000. This approach can allow initial alignment to be accurate and stable in less than 5 min at any sea conditions by examining the apparent movement of gravity in a fixed inertial frame. This initial alignment approach is used in the advanced Octans system products of the IxSea Company. Perhaps due to the protection of technical secrets, only a brief description of the approach is presented in [7], so unfortunately, we cannot obtain sufficient technical details for implementing this initial alignment approach. Originating from the basic idea of this alignment approach, many research studies on the initial alignment using the projection of gravity in the inertial frame have been presented since then [8,9,10,11,12,13,14,15,16].

From the perspective of the principle of alignment, the current research can be classified into two categories: approaches based on the indirect and direct use of the movement of gravity. In the indirect method [8,9,10,11,12,13], the initial attitude matrix is decomposed into the product of three attitude matrices. The difficulty is to determine one of them, that is, an attitude matrix between the two defined frames which are fixed frames in the inertial space. To determine the attitude matrix by solving Wahba’s problem, two non-collinear vectors are constructed by the integrations of the projection of the gravity into the two defined frames over one period, and another two non-collinear vectors are generated by the integrations over another period. The results demonstrate that this approach can be applied for marine coarse alignment. An improved indirect method is proposed in [9] to decrease the variance of the alignment error.

The movement of gravity is embodied in the two non-collinear vectors, which is indirectly used in the alignment algorithm in [8,9,10,11,12,13]. However, this indirect method is complicated since it involves five reference frames and three attitude matrices in total. Furthermore, accurate latitude and longitude information is necessary for this method to calculate the final alignment results; that is to say, it is not a self-alignment method. The other alignment approach [14,15,16], namely the direct method, has none of these drawbacks.

In the direct method [14,15,16], the initial attitude matrix is decomposed into two attitude matrices. One is the attitude matrix from the body frame to the inertial frame. It can be obtained by the integral of the angular velocity measured by gyroscopes. The other is the attitude matrix from the inertial frame to the navigation frame. The gravity vector in the inertial frame and its derivative with time are directly used to calculate this attitude matrix. This direct method is more intuitional than the indirect method in [8,9,10,11,12,13]. The alignment algorithm is also more concise since only three frames and two attitude matrices are involved. Furthermore, it is a self-alignment without the dependence of any external information such as latitude and longitude.

For the direct method, an important but difficult task is to obtain the derivative of the gravity vector. In the current research [14,15,16], the approximate derivative of gravity is obtained by a difference operation based on discrete gravity measurements at different moments. In principle, exact data are required for the numerical differential procedures to obtain desirable results. Otherwise even a slight data error can lead a substantially large error in the numerical differential results [17,18]. However, the actual measurements inevitably contain types of error sources including offset, random noise, quantization error, etc. To solve this problem, [14,15] applied a low-pass filter while [16] used parameter identification and reconstruction to pre-process the measurement data.

Although adopting the preprocessing measures of data can achieve good results, it is useless for resolving the intrinsic problem of the numerical differential for non-exact data, which is a typical inverse problem [19]. The presence of the numerical differential is a hidden danger and can cause substantial error in the accuracy and stability of alignment results in practical applications.

In this paper, a novel alignment approach for marine strapdown INS is proposed. We follow the basic idea of the direct method [14,15,16], so our approach can provide a concise self-alignment algorithm. In terms of calculating the derivative of gravity, an innovative method is presented based on the trajectory fitting of the apparent movement of gravity in the inertial frame. Through the trajectory fitting, we can obtain the analytic expressions of the derivative of gravity so that the numerical differential can be abandoned.

The major contribution of this paper is that we propose a radically different method from the current research to calculate the derivative of gravity for the self-alignment. Consequently, the challenges of the numerical differential in calculating the derivative of gravity are fundamentally addressed. The details of the novel method are presented, and the results from the simulation and the experimental test verify the effectiveness of this novel algorithm.

The rest of this paper is organized as follows. In Section 2, the definitions of the frames used in this paper are presented. The general principle of the coarse alignment is explained in Section 3. Section 4 details the fitting of the spatial circle, namely the trajectory of gravity movement in the inertial frame. In Section 5, the determination of the final attitude matrix is presented. Experimental results and analyses are presented in Section 6. Finally, conclusions are summarized in Section 7.

## 2. Frame Definitions

The reference frames used in this paper are defined as follows [20]:

(1) The navigation frame (n-frame $OX_{n}Y_{n}Z_{n}$): this frame is a local geographic frame, whose origin *O* is set at the ship location, and its axes are aligned with the directions of North, East, and the local vertical (Up).
(2) The body frame (b-frame $OX_{b}Y_{b}Z_{b}$): this frame is an orthogonal axis set, whose origin is the mass center of the ship, and its axes are aligned with the roll, pitch, and yaw axes of the ship.
(3) The inertial frame (i-frame $OX_{i}Y_{i}Z_{i}$): this frame is the b-frame at initial time $t_{0}$. After starting the system, this frame remains fixed, which belongs to the inertial space.

**Figure 1:**
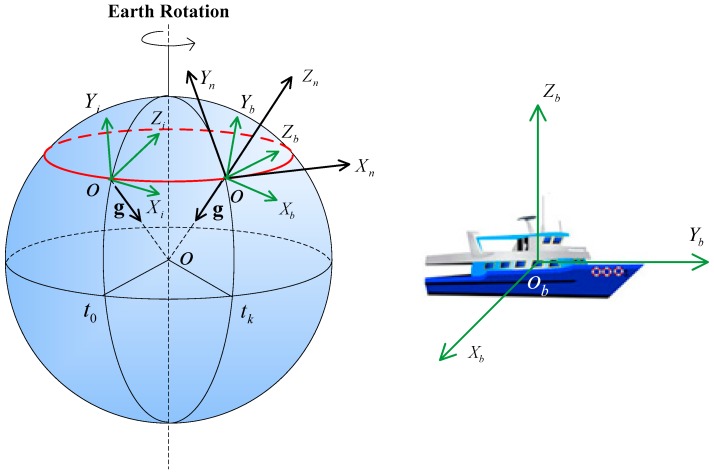
Frame definitions and the movement of gravity in the inertial space.

The frames mentioned above are shown in Figure 1.

## 3. Principle of the Coarse Alignment

As shown in Figure 1, the gravity vector expressed in the inertial frame (i-frame) changes with time. A vector in a frame coordinate is denoted by a point. The trajectory of the movement of gravity in the i-frame is a circle due to the rotation of the Earth, which is represented by the red circle. By observing the movement of gravity, we can determine the attitude matrix between the i-frame and the n-frame.

**Figure 2:**
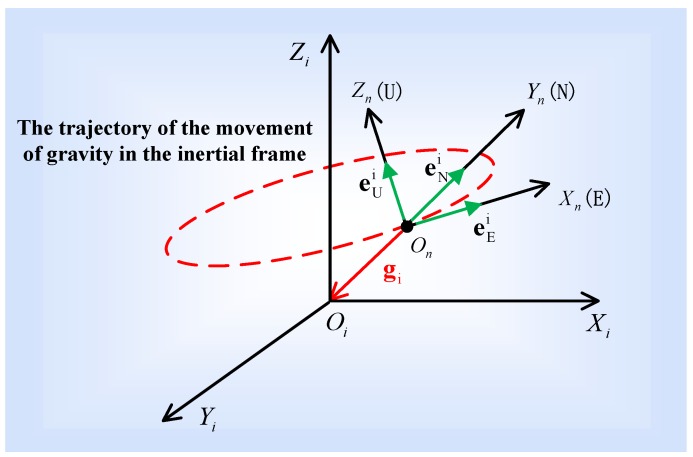
The relation between the i-frame and the n-frame by using the gravity vector.

Figure 2 shows the relation between the i-frame and n-frame by using the gravity vector. As seen in Figure 2, the Up axis is parallel to the gravity vector expressed in the i-frame, the East axis lies along the derivative of the gravity vector, and the North axis is the vector product of the Up and East axes. $e_{U}^{i}$ represents the direction of the Up axis in the i-frame, which is a 3-dimensional (3-D) unit vector along the Up axis by normalization. $e_{E}^{i}$ represents the direction of the East axis in the i-frame, which is a 3-D unit vector along the East axis by normalization. $e_{N}^{i}$ represents the direction of the North axis in the i-frame, which is a 3-D unit vector along the North axis by normalization.

Based on the above analyses and Figure 2, the unit vectors of the Up, East, and North axis expressed within the i-frame can be formalized by

(1) $$\left\{ \begin{array}{l} e_{U}^{i}=-\frac{g_{i}}{\left\|g_{i}\right\|}\\ e_{E}^{i}=-\frac{dg_{i}}{dt}/\left\|\frac{dg_{i}}{dt}\right\|\\ e_{N}^{i}=i_{U}\times i_{E} \end{array}\right.$$

where, $g_{i}$ is the gravity vector expressed in the i-frame, and ‖‖ represents the calculation of vector module.

It is easy to see that the unit vectors of the Up, East, and North axis expressed within the n-frame is an identical matrix, so we can obtain the following relationship between the unit vector of the Up, East, and North axis expressed within the i-frame and the n-frame

(2) $$C_{i}^{n}[\begin{array}{ccc} e_{E}^{i} & e_{N}^{i} & e_{U}^{i} \end{array}]=[\begin{array}{ccc} e_{E}^{n} & e_{N}^{n} & e_{U}^{n} \end{array}]=\left[\begin{array}{ccc} 1 & 0 & 0\\ 0 & 1 & 0\\ 0 & 0 & 1 \end{array}\right]$$

$C_{i}^{n}$ is the attitude matrix between the i-frame and the n-frame, which can then be computed by

(3) $$C_{i}^{n}={\left[\begin{array}{ccc} e_{E}^{i} & e_{N}^{i} & e_{U}^{i} \end{array}\right]}^{-1}$$

The gyroscopes can sense the b-frame’s angular velocity expressed in the i-frame, which is denoted by $\omega_{\mathrm{ib}}^{b}$. The attitude matrix $C_{b}^{i}$ can be calculated by solving the following differential equation

(4) $${\dot{C}}_{b}^{i}=C_{b}^{i}(\omega_{\mathrm{ib}}^{b}\times )$$

where $\left(\omega_{\mathrm{ib}}^{b}\times \right)$ is the skew symmetric cross-product matrix of $\omega_{\mathrm{ib}}^{b}$. According to the definition of the i-frame, at the initial time the b-frame coincides with the i-frame, so the initial $C_{b}^{i}$ is $I_{3\times 3}$.

While the ship is moored at sea for the initial alignment, the accelerometers can measure the sum of the gravity and interferential acceleration. After filtering out the interferential acceleration, we obtain the gravity in b-frame, $g_{b}$, from the accelerometer measurements $f_{b}$ namely $f_{b}=-g_{b}$. Combined with the attitude matrix $C_{b}^{i}$, the gravity expressed in the i-frame $g_{i}$ can be written as

(5) $$g_{i}=-C_{b}^{i}f_{b}$$

In terms of filtering out the interferential acceleration, many researchers have studied and provided various approaches for alignment applications, for instance finite impulse response (FIR) low-pass digital filters in [14,15], a Hidden Markov model based Kalman filter in [10], and a recognition and reconstruction algorithm for gravity movement in [16]. There is no doubt that the filtering is necessary in this alignment application and a better performance of the filter can provide better alignment results. In this paper, we propose an improved alignment method, which has a lower sensitivity to the acceleration noise. The method of moving average is applied here for pre-processing the IMU measurements.

By substituting the $g_{i}$ and its derivative into Equation (1), the $C_{i}^{n}$ can be obtained by using Equations (1) and (3). Combining that with the $C_{b}^{i}$ from Equation (4), the coarse attitude matrix between the b-frame and the n-frame can be determined by Equation (6), and thus the initial alignment can be completed.

(6) $$C_{b}^{n}=C_{b}^{i}C_{i}^{n}$$

The rocking disturbances are contained in the gyroscope measurements. In traditional coarse alignment [2,3,4], the disturbances were expected to be eliminated. However, for the proposed approach, all the gyroscope measurements are needed as shown in Equations (4–6). From the perspective of principles, rocking disturbances cannot contaminate the alignment results. Therefore, the coarse alignment directly using the gravity can be suitable for marine strapdown INS.

**Figure 3:**
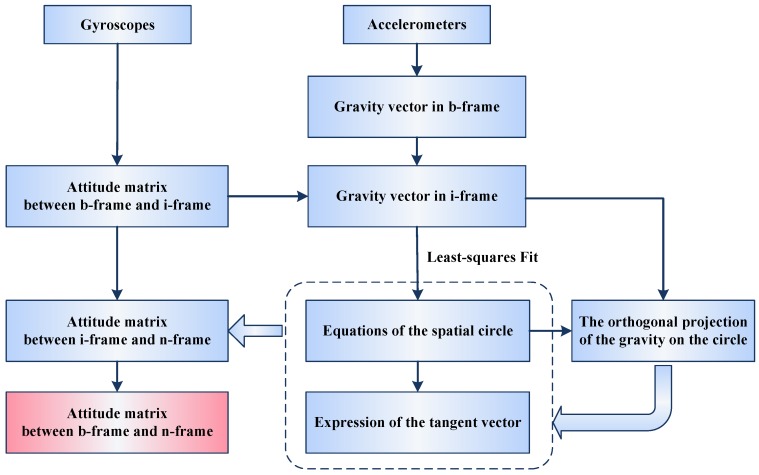
The general process of the coarse alignment.

To solve for the difficulty in calculating the derivative of gravity as explained in Section 1, a fitting method to the trajectory of the gravitational movement was developed. The general principle of the approach is summarized in Figure 3.

The trajectory of the gravity movement in the inertial frame is a circle, as illustrated in Figure 1 and Figure 2. Therefore, the derivative of gravity is the tangent vector of the gravity on the circle. We first calculate the gravity expressed in the inertial frame by the IMU measurements. Then, a best fit of the circle to the gravity data is obtained by a least-squares method. The fitting can provide analytical expressions of the spatial circle. According to the expressions, we can derive the tangent vector of any point on the circle, i.e., the derivative of the gravity vector. Based on the accurate expression of the circle, this approach to calculating the gravity’s derivative is robust while using noisy data. The details are presented in the following sections.

## 4. Least-Squares Fitting of the Spatial Circle

Least-squares fitting was used to estimate the optimal parameterized model of the fitted curve (or surface) by minimizing the sum of the square of geometric distances from the observed data to the fitted curve (or surface) [21].

The general model of a spatial circle can be expressed by the following system of equations.

(7) $$\{\begin{array}{c} Ax+By+Cz+D=0\\ {\left(x-x_{0}\right)}^{2}+{\left(y-y_{0}\right)}^{2}+{\left(z-z_{0}\right)}^{2}=R^{2} \end{array}$$

where Ax+By+Cz+D=0 represents the plane that the circle lies on, and (A,B,C) is the normal vector of the plane. ${\left(x-x_{0}\right)}^{2}+{\left(y-y_{0}\right)}^{2}+{\left(z-z_{0}\right)}^{2}=R^{2}$ represents the sphere surface that the circle belongs to. $\left(x_{0},y_{0},z_{0}\right)$ and R are the center and radius of the sphere, respectively.

For the fitting of the circle, (A,B,C,D) and $\left(x_{0},y_{0},z_{0},R\right)$ are the parameters to be estimated by the least-squares method. Considering the accuracy and robustness of the fitting results as well as practical implementation, we designed the following two-step processes for the fitting of the spatial circle.

1. Based on the data of gravity $g_{i}$ offered by Equation (5), the least-squares fitting of the plane is first accomplished.
2. The center of the sphere is approximately located at the plane, which generates a linear equality constraint for parameters $\left(x_{0},y_{0},z_{0}\right)$. The sphere is then fitted by a constrained least-squares method.

### 4.1. Plane Fitting

The purpose of the plane fitting is to estimate the parameters *A*, *B*, *C*, and *D* in Equation (7). Using 3-D data, we can obtain

(8) $$\left[\begin{array}{cccc} x_{1} & y_{1} & z_{1} & 1\\ x_{2} & y_{2} & z_{2} & 1\\ \vdots & \vdots & \vdots & \vdots \\ x_{n} & y_{n} & z_{n} & 1 \end{array}\right]\left[\begin{array}{c} A\\ B\\ C\\ D \end{array}\right]=\left[\begin{array}{c} 0\\ 0\\ \vdots \\ 0 \end{array}\right]$$

where $\left(x_{1},y_{1},z_{1}\right)$, $\left(x_{2},y_{2},z_{2}\right)$, …, $\left(x_{n},y_{n},z_{n}\right)$ are the observed gravity data in Equation (5), and n represents the amount of the data. Equation (8) can be rewritten in the matrix form

(9) Ma=0

where

(10) $$M=\left[\begin{array}{cccc} x_{1} & y_{1} & z_{1} & 1\\ x_{2} & y_{2} & z_{2} & 1\\ \vdots & \vdots & \vdots & \vdots \\ x_{n} & y_{n} & z_{n} & 1 \end{array}\right]a={\left[\begin{array}{cccc} A & B & C & D \end{array}\right]}^{T}$$

Equation (9) is a homogeneous and overdetermined system of equations, in which a is the unknown to be solved. To compute its least-squares solution, the problem can be described as [22]

(11) $$\left\{ \begin{array}{c} \min\;{\left\|Ma\right\|}^{2}\\ \mathrm{subject}\;\mathrm{to}\;\left\|a\right\|=1 \end{array}\right.$$

where the objective function ${\left\|Ma\right\|}^{2}$ equals to

(12) $${\left\|Ma\right\|}^{2}=a^{T}(M^{T}M)a=\lambda \left\|a\right\|=\lambda$$

where λ is the eigenvalue of $M^{T}M$. The problem expressed by Equation (11) then transforms to the calculation of the minimal eigenvalue. Thus, the least-squares solution is the eigenvector corresponding to the smallest eigenvalue. We apply singular value decomposition (SVD) of M to compute the smallest eigenvector.

**M** is decomposed using SVD into

(13) $$M=USV^{T}$$

where **S** is a diagonal matrix of the singular values of M, and U and V are the left-hand and right-hand eigenvector matrices, respectively. The least-squares solution of a is the singular vector in V corresponding to the smallest singular value.

### 4.2. Sphere Fitting

Determining the optimal fit of a sphere is a nonlinear least-squares problem, which may be solved by iterative minimization algorithms, such as Gauss-Newton or Levenberg-Marquardt [23]. The two methods normally converge in 5–10 iterations [24], but they are extremely sensitive to the presence of outliers. This approach is impractical due to its occasional divergence. Alternatively, we employed an approach that can reduce the problem to a linear least-squares problem. This approach has advantages in its implementation and computing costs [25].

We generalized the circle fitting by linear least squares in [23] to the sphere fitting. To find the best fit for a sphere in a least-squares sense to estimate the parameters $x_{0}$, $y_{0}$, $z_{0}$ and *R* in Equation (7), we can solve the problem

(14) $$\min\;\sum_{i=1}^{n}\left\{f_{i}(x_{0},y_{0},z_{0},R\right){\}}^{2}$$

where $f(x_{0},y_{0},z_{0},R)$ is the residual described as

(15) $$f_{i}(x_{0},y_{0},z_{0},R)={\left(x_{i}-x_{0}\right)}^{2}+{\left(y_{i}-y_{0}\right)}^{2}+{\left(z_{i}-z_{0}\right)}^{2}-R^{2}$$

At first sight, this problem is a nonlinear least-squares problem. However, $f_{i}(x_{0},y_{0},z_{0},R)$ can be written in a linear form by changing the parameters. Therefore, Equation (15) can be expanded as

(16) $$f_{i}(x_{0},y_{0},z_{0},R)=-2x_{i}x_{0}-2y_{i}y_{0}-2z_{i}z_{0}+x_{0}^{2}+y_{0}^{2}+z_{0}^{2}-R^{2}+x_{i}^{2}+y_{i}^{2}+z_{i}^{2}$$

letting

(17) $$X_{0}=2x_{0},\;Y_{0}=2y_{0},\;Z_{0}=2z_{0},\;N=x_{0}^{2}+y_{0}^{2}+z_{0}^{2}-R^{2}$$

then the problem expressed by Equation (14) becomes

(18) $$\min\;\sum_{i}^{n}\{-x_{i}X_{0}-y_{i}Y_{0}-z_{i}Z_{0}+N+x_{i}^{2}+y_{i}^{2}+z_{i}^{2}{\}}^{2}$$

or is expressed in a compact form of matrix

(19) $$\min{\left\|X\beta -y\right\|}^{2}$$

where

(20) $$X=\left[\begin{array}{cccc} x_{1} & y_{1} & z_{1} & 1\\ x_{2} & y_{2} & z_{2} & 1\\ \vdots & \vdots & \vdots & \vdots \\ x_{n} & y_{n} & z_{n} & 1 \end{array}\right],\;\beta =\left[\begin{array}{c} X_{0}\\ Y_{0}\\ Z_{0}\\ N \end{array}\right],\;y=\left[\begin{array}{c} x_{1}^{2}+y_{1}^{2}+z_{1}^{2}\\ x_{2}^{2}+y_{2}^{2}+z_{2}^{2}\\ \vdots \\ x_{n}^{2}+y_{n}^{2}+z_{n}^{2} \end{array}\right]$$

By transforming the parameters from $\left(x_{0},y_{0},z_{0},R\right)$ to $\left(X_{0},Y_{0},Z_{0},N\right)$, problem expressed by Equation (14) changes to a simple linear least-square problem expressed by Equation (19) which can be readily solved. 

In addition, considering that the sphere center lies on the fitted plane in Section 4.1, the parameters $\left(x_{0},y_{0},z_{0}\right)$ satisfy the following linear equality

(21) $$Ax_{0}+By_{0}+Cz_{0}+D=0$$

Based on Equations (17) and (21), we can obtain

(22) Wβ=d

where

(23) $$W=\left[\begin{array}{cccc} \frac{A}{2} & \frac{B}{2} & \frac{C}{2} & 0 \end{array}\right],\;d=-D$$

Introducing the linear equality (21) as a constraint to the linear least-squares problem can improve the accuracy and robustness of the sphere fitting. The constrained least-squares problem is described by

(24) $$\{\begin{array}{c} \min{\left\|X\beta -y\right\|}^{2}\\ \mathrm{subject}\;\mathrm{to}\;W\beta =d \end{array}$$

There are several algorithms for solving the linear least squares with linear equality constraints [26]. We utilize the algorithms using the Lagrangian multiplier method in [27], and then the solution to the problem expressed by Equation (24) is

(25) $$\beta ={\left(X^{T}X\right)}^{-1}X^{T}y-{\left(X^{T}X\right)}^{-1}W^{T}{\left[W{\left(X^{T}X\right)}^{-1}W^{T}\right]}^{-1}\times [W{\left(X^{T}X\right)}^{-1}X^{T}y-d]$$

After estimating β, Equation (17) shows that the parameters $\left(x_{0},y_{0},z_{0},R\right)$ are equal to

(26) $$x_{0}=\frac{X_{0}}{2},\;y_{0}=\frac{Y_{0}}{2},\;z_{0}=\frac{Z_{0}}{2},\;R=\sqrt{x_{0}^{2}+y_{0}^{2}+z_{0}^{2}-N}$$

Substituting the parameters a and β estimated in Section 4.1 and Section 4.2 into Equation (7), we can determine a best fit of the spatial circle to the observed gravity data in a least-squares sense.

## 5. Determination of the Initial Attitude Matrix

### 5.1. Projection of the Measured Gravity Data on the Fitted Circle

The fitted circle approximates the original gravity data in Equation (5), which is denoted by $\left(x_{i},y_{i},z_{i}\right)$. The original data is noisy due to the IMU noise. It is required to know which point on the circle corresponds to the original data for two reasons. First, the point on the fitted circle is better than the original data in terms of the error variance. Thus, using the data on the circle instead of the original data is more accurate for determining the Up axis in Equation (1). Second, an exact point on the circle is needed to determine the East axis, by being substituted into the subsequent Equations (32) and (33). According to the least-squares criterion, the original data should correspond to its orthogonal projection point on the circle. The orthogonal projection of the original data onto the circle can be summarized by three steps.

1. Calculate the orthogonal projection point $\left(x_{i}^{\prime },y_{i}^{\prime },z_{i}^{\prime }\right)$ onto the plane Ax+By+Cz+D=0 fitted in Section 4 by

(27) $$\{\begin{array}{c} x_{i}^{\prime }=Ak+x_{i}\\ y_{i}^{\prime }=Bk+y_{i}\\ z_{i}^{\prime }=Ck+z_{i} \end{array}$$

where

(28) $$k=\frac{-(Ax_{i}+By_{i}+Cz_{i}+D)}{A^{2}+B^{2}+C^{2}}$$

2. Provide the equation of the straight line through $\left(x_{i}^{\prime },y_{i}^{\prime },z_{i}^{\prime }\right)$ and the circle center $\left(x_{0},y_{0},z_{0}\right)$, which can be described by

(29) $$\frac{\left(x-x_{0}\right)}{\left(x_{i}^{\prime }-x_{0}\right)}=\frac{\left(y-y_{0}\right)}{\left(y_{i}^{\prime }-y_{0}\right)}=\frac{\left(z-z_{0}\right)}{\left(z_{i}^{\prime }-z_{0}\right)}$$

Then rewrite this line equation in the following form

(30) $$\{\begin{array}{c} (y_{i}^{\prime }-y_{0})x-(x_{i}^{\prime }-x_{0})y+[(x_{i}^{\prime }-x_{0})y_{0}-(y_{i}^{\prime }-y_{0})x_{0}]=0\\ (z_{i}^{\prime }-z_{0})y-(y_{i}^{\prime }-y_{0})z+[(y_{i}^{\prime }-y_{0})z_{0}-(z_{i}^{\prime }-z_{0})y_{0}]=0 \end{array}$$

3. Solve the system of equations ${\left(x-x_{0}\right)}^{2}+{\left(y-y_{0}\right)}^{2}+{\left(z-z_{0}\right)}^{2}=R^{2}$ and Equation (30). Two intersection points can be obtained. Because the center $\left(x_{0},y_{0},z_{0}\right)$ belongs to the fitted plane, the straight line also intersects with the fitted circle. Hence, the intersection point near $\left(x_{i}^{\prime },y_{i}^{\prime },z_{i}^{\prime }\right)$ has the shortest distance to $\left(x_{i}^{\prime },y_{i}^{\prime },z_{i}^{\prime }\right)$ as compared with the other points on the circle; that is to say, it is the orthogonal projection of the original data $\left(x_{i},y_{i},z_{i}\right)$ onto the fitted circle.

### 5.2. Determination of the Initial Attitude Matrix

Instead of the original data, the orthogonal projection point represents the gravity vector in the i-frame, which is denoted by $g_{i}$. We can directly apply $g_{i}$ to Equation (1) to determine the direction of the Up axis in the i-frame, namely $e_{U}^{i}$.

As illustrated in Section 3, the East axis in the i-frame is parallel to the direction of the tangent vector of $g_{i}$ on the fitted circle. We first derive the expression of the tangent vector of any point on the circle described by Equation (7).

For the system described by Equation (7), assume x is an independent variable, and y and z are dependent variables on x (one also can assume y or z is the independent variable). Then, by deriving the derivatives with respect to x in the system described by Equation (7), we can obtain

(31) $$\left\{ \begin{array}{l} \left(y-y_{0})\frac{dy}{dx}+(z-z_{0})\frac{dz}{dx}=-(x-x_{0}\right)\\ B\frac{dy}{dx}+C\frac{dz}{dx}=-A \end{array}\right.$$

Solving this system of equations, the partial derivative of y and z with respect to x can be obtained, which are

(32) $$\left\{ \begin{array}{l} \frac{dy}{dx}=\frac{A(z-z_{0})-C(x-x_{0})}{C(y-y_{0})-B(z-z_{0})}\\ \frac{dz}{dx}=\frac{B(x-x_{0})-A(y-y_{0})}{C(y-y_{0})-B(z-z_{0})} \end{array}\right.$$

Then, the tangent vector E of any point on the circle is

(33) $$E=\left(\begin{array}{ccc} 1 & \frac{dy}{dx} & \frac{dz}{dx} \end{array}\right)$$

The unit vector of the East axis in the i-frame, namely $e_{E}^{i}$ in Equation (1), can be obtained by the normalization of E.

By using $e_{U}^{i}$ and $e_{E}^{i}$ obtained here, the initial attitude matrix can be determined by Equations (1)–(6).

Our coarse alignment approach can benefit the alignment performance in three aspects. First, rather than using the original gravity measurements in the i-frame, we use their orthogonal projection onto the fitted circle as the gravity vector. The noise of the original data can be hence eliminated to some extent, which can result in an improvement in the final alignment accuracy. Second, instead of numerical differentiation, the expression of the tangent vector is used to calculate the derivative of the gravity vector. This method can desensitize the results to data noise and hence lead to good robustness. Third, the accurate equations of the circle allow the alignment results to be stable for repeated experiments.

## 6. Experimental Results and Analyses

### 6.1. Coarse Alignment Using Simulated Data

To verify the performance of the proposed coarse alignment approach, simulations were conducted at three typical sea conditions (calm, moderate, and severe sea conditions). The simulation conditions were set as follows.

(1) Rocking motion

The resulting attitude, i.e., pitch, roll, and heading angle, from the sea waves were modeled as the following sine function

amplitude⋅sin(2πt/period)

■ In the case of calm sea condition, the amplitude/period were set as
  pitch:5°/10 s  roll:4°/8 s  heading:5°/12 s
■ In the case of moderate sea condition, the amplitude/period were set as
  pitch:10°/5 s  roll:6°/6 s  heading:8°/7 s
■ In the case of severe sea condition, the amplitude/period were set as
  pitch:15°/2 s  roll:20°/3 s  heading:20°/3 s

(2) Linear motion

The ship was simulated to be moored and the velocity was set to zero.

(3) IMU biases 

The offset of the gyroscopes was set as 0.01°/h, and the random white noise was set as 0.001°/h standard deviation (SD). The offset of the accelerometers was set as $10^{-4}\;g$, and the random white noise was set as $10^{-5}g$ SD.

The time of coarse alignment is 5 min, and the simulation step was 0.01 s. The generated acceleration (gravity) measurements were preprocessed by moving average for filtering out acceleration biases. The acceleration data was sampled every 10 s. Then, 30 sets of acceleration data were used for the circle fitting. For each sea condition, we conducted the simulation of coarse alignment 50 times. The obtained alignment results were used to verify the practical performance of the alignment. The results of our coarse alignment at the three sea conditions are shown below.

**Figure 4:**
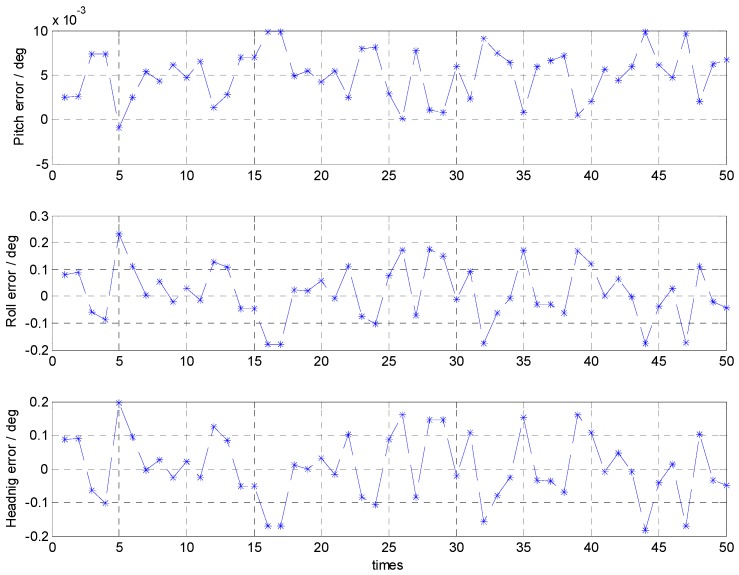
Attitude errors of the coarse alignment with the calm sea condition.

With the calm sea condition, the errors of the attitude alignment are shown in Figure 4 and their statistical analyses are listed in Table 1. In terms of the statistical analyses, the maximum and minimum of the alignment error results can reflect the alignment accuracy, and the standard deviation is related to the stability of the alignment results.

The results show that, with the calm sea condition, the attitude error is less than 0.25° and its maximum standard deviation is about 0.1°.

**Figure 5:**
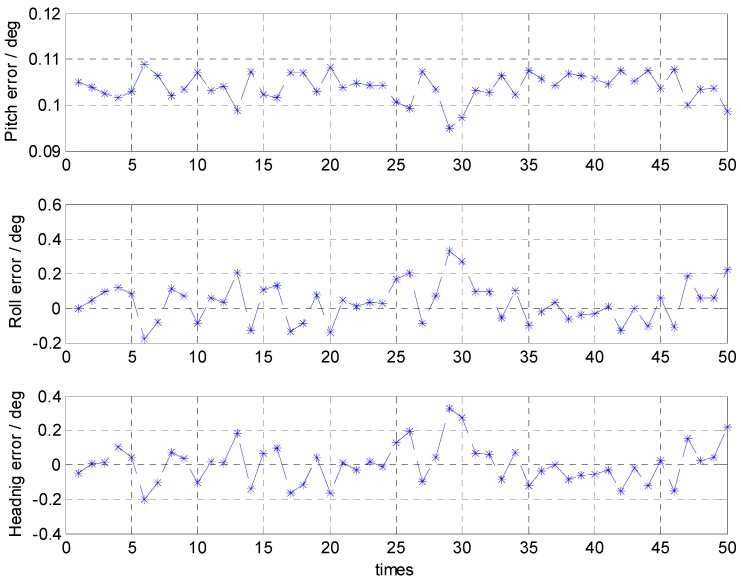
Attitude errors of the coarse alignment with the moderate sea condition.

With the moderate sea condition, the errors of the attitude alignment are shown in Figure 5 and their statistical analyses are listed in Table 2.

The results show that with the moderate sea condition, the attitude error is less than 0.34° and its maximum standard deviation is about 0.1°.

**Figure 6:**
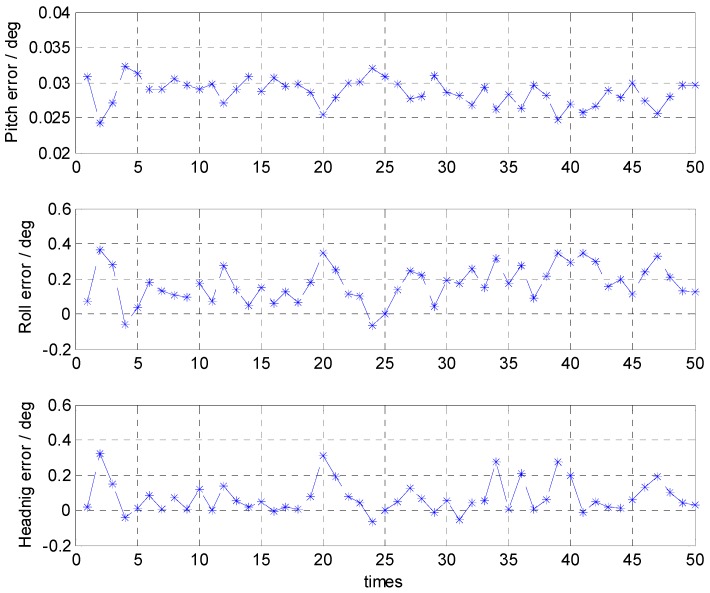
Attitude errors of the coarse alignment with the severe sea condition.

With the severe sea condition, the errors of the attitude alignment are shown in Figure 6 and their statistical analyses are listed in Table 3.

The results show that with the severe sea condition, the attitude error is less than 0.37° and its maximum standard deviation is about 0.1°.

The results from the 50 simulation runs showed that the attitude errors of coarse alignment were less than 0.37° and the mean values were roughly a few minutes of arc with the three sea conditions. Additionally, the standard deviation of pitch error, roll error, and heading error are about 0.002°, 0.1°, and 0.1°, respectively.

It can also be seen that the maximum attitude error increases with the sea condition. With the severe sea condition, the large amplitude and high frequency of the attitude angle can enlarge the effect of the accelerometers’ biases on the alignment error. Therefore, it is necessary to eliminate the acceleration disturbances caused by the rocking for practical applications.

The results imply that our coarse alignment approach is capable of being employed with any sea conditions. Additionally, the coarse alignment is significantly accurate and stable for marine applications.

### 6.2. Quasi-Static Alignment Using Trial Data

**Figure 7:**
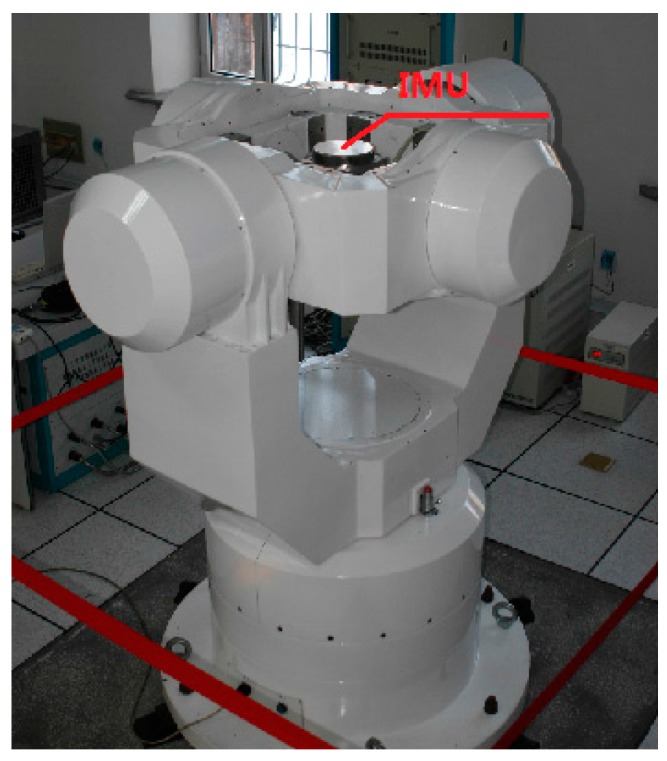
Three-axis turntable for experiments.

In addition to the simulations, practical experiments were conducted to test the performance of the coarse alignment. A moderate-precision IMU and a SGT-3 three-axis turntable were used for the experiments. The IMU was mounted on the turntable, as shown in Figure 7. The offset of the gyroscopes was 0.003°/h, and the random white noise was $2.5\times 10^{-4}\;{}^{\circ}/h$ SD. The offset of the accelerometers was $10^{-5}\;g$, and the random white noise was $5\times 10^{-6}\;g$ SD.

**Figure 8:**
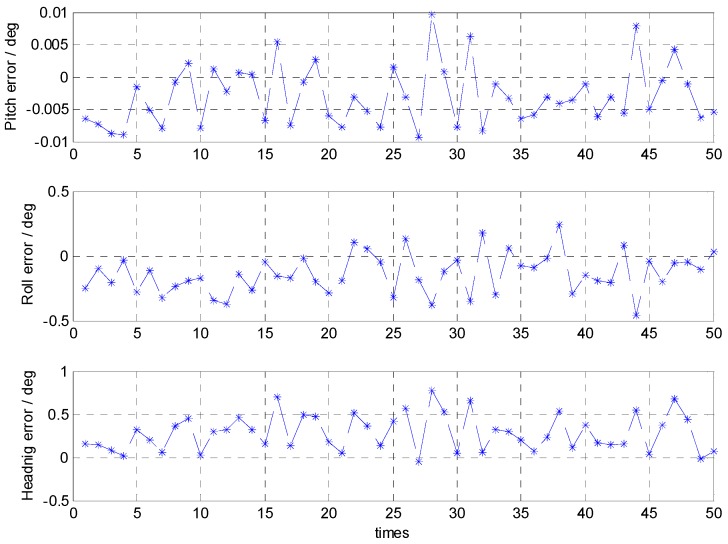
Attitude errors of the coarse alignment using the trial data.

The coarse alignment lasted 5 min and sampling time was 0.01 s. During the coarse alignment, the turntable was quasi-static. We collected 50 sets of the measured data by IMU for testing the coarse alignment. For the turntable, an exact reference of the attitude was known. At the end of the alignment time, the attitude errors based on the 50 sets of trial data were determined and are shown in Figure 8. Their statistical analyses are presented in Table 4.

The results based on the trial data showed that the attitude errors of coarse alignment are less than 0.8° and the mean values are roughly ten minutes of arc. Additionally, the standard deviation of pitch error, roll error, and heading error were about 0.004°, 0.15°, and 0.21°, respectively. This performance satisfied the requirements of accuracy and stability, and demonstrated that the proposed coarse alignment can be applied for marine strapdown INS.

## 7. Conclusions

Here we have presented an innovative approach to coarse alignment for marine strapdown INS. The detailed approach is distinguished from that in the current published literature, which can address the difficulty in calculating the derivative of gravity. By using the trajectory fitting of the gravity movement in the inertial frame, we can examine the movement of gravity. The movement of the gravity vector can express the attitude matrix between the navigation frame and the inertial frame. The integration of the angular velocity measured by gyroscopes can provide the attitude matrix between the body frame and the inertial frame. Subsequently, the initial attitude matrix can be determined without any external information. This approach to coarse alignment allows the INS to function despite severe rocking disturbances. The experimental results have demonstrated that the coarse alignment can provide accurate and stable results with any sea conditions.

## Figures and Tables

**Table 1:** Statistical analyses of the attitude errors with the calm sea condition.

	Pitch Error (°)	Roll Error (°)	Heading Error (°)
Mean	0.0051	0.0130	0.0042
STD	0.0028	0.1026	0.0984
Max	0.0099	0.2317	0.1964
Min	−0.0009	−0.1784	−0.1828

**Table 2:** Statistical analyses of the attitude errors with the moderate sea condition.

	Pitch Error (°)	Roll Error (°)	Heading Error (°)
Mean	0.1040	0.0315	0.0051
STD	0.0030	0.1145	0.1158
Max	0.1089	0.3318	0.3320
Min	0.0950	−0.1785	−0.2022

**Table 3:** Statistical analyses of the attitude errors with the severe sea condition.

	Pitch Error (°)	Roll Error (°)	Heading Error (°)
**Mean**	0.0286	0.1698	0.0726
**STD**	0.0019	0.1053	0.0914
**Max**	0.0324	0.3631	0.3242
**Min**	0.0242	−0.0682	−0.0632

**Table 4:** Statistical analyses of the attitude errors using the trial data.

	Pitch Error (°)	Roll Error (°)	Heading Error (°)
**Mean**	−0.0030	−0.1369	0.2839
**STD**	0.0046	0.1542	0.2114
**Max**	0.0096	0.2442	0.7821
**Min**	−0.0093	−0.4588	−0.0496
